# Sharing, reuse, and storage of biosamples among biomedical researchers in Jordan: Practice and concerns

**DOI:** 10.1371/journal.pone.0267552

**Published:** 2022-04-28

**Authors:** Almuthanna K. Alkaraki, Omar F. Khabour, Karem H. Alzoubi, Lina M. K. Al-Ebbini, Zaid Altaany

**Affiliations:** 1 Department of Biological Sciences, Faculty of Science, Yarmouk University, Irbid, Jordan; 2 Department of Medical Laboratory Sciences, Faculty of Applied Medical Sciences, Jordan University of Science and Technology, Irbid, Jordan; 3 Department of Pharmacy Practice and Pharmacotherapeutics, University of Sharjah, Sharjah, UAE; 4 Department of Clinical Pharmacy, Faculty of Pharmacy, Jordan University of Science and Technology, Irbid, Jordan; 5 Department of Biomedical Systems and Informatics Engineering, Hijjawi Faculty for Engineering Technology, Yarmouk University, Irbid, Jordan; 6 Department of Basic Medical Sciences, Faculty of Medicine, Yarmouk University, Irbid, Jordan; Imam Abdulrahman Bin Faisal University, SAUDI ARABIA

## Abstract

**Background:**

Sharing and reuse biosamples can facilitate biomedical research. Little is known about researchers’ perception and practice about sharing, reusing, and storing biosamples in Jordan. Therefore, the current study aimed to evaluate the practices of biomedical researchers in Jordan regarding biosamples management.

**Methods:**

The study was cross-sectional and involved biomedical researchers from different parts of Jordan. A questionnaire was designed to achieve the aim of this study. The questionnaire was web-based and distributed via e-mails using Google forms.

**Results:**

Opinions of Jordanian biomedical researchers from different academic ranks and institutional backgrounds were measured and recorded anonymously. The majority of the sample was males (57.9%), from public universities (64.3%), and (64.6%) were from health-related fields. About 82.9% of participants stored biosamples using codes, whereas the rest used the subject’s name. Sharing of biosamples was commonly practiced by 61.7% of Jordanian researchers locally, while 47.2% of the Jordanian researchers shared biosamples overseas. The reuse of biosamples in other projects was reported to be 55.4%. The majority explained the possibility of reusing and sharing biosamples in the consent form (range: 53–58%). Sharing and reusing biosamples were associated with gender, the number of publications in peer-reviewed international journals, and academic rank (P<0.05).

**Conclusion:**

Sharing and reusing biosamples are common among Jordanian biomedical researchers. Therefore, ethically grounded biosamples sharing and reuse standards are essential for protecting human subjects’ rights and privacy in Jordan.

## Introduction

Sharing and reusing biosamples are helpful for biomedical researchers, where their reuse and repurposing can exploit their value and accelerate scientific discoveries and excellence [1]. Such practices can save the time and efforts of subject recruitment and avoid pain and discomfort during the sampling process [2]. Moreover, sharing and reusing biosamples could validate results by using higher sample size, improving statistical analysis, and help in understanding different biological mechanisms of rare diseases [3, 4].

The benefits of sharing and reusing biosamples should not take precedence over the rights of human subjects such as autonomy and privacy yet during the process of biosamples sharing and reusing, both autonomy and privacy of subject may be affected [5, 6]. Sharing of biosamples could reveal the donor identity, thus breaching confidentiality, with the possibility of discrimination [1, 7–10]. The ethical issues related to the sharing and reusing of biological samples came to the front with the rise of biobanks. It is suggested that biobanks play a vital role in biomedical research involving human subjects and provide crucial input to the rapid growth of scientific efforts [11]. The importance of biobanks relies on the ability of researchers to reuse biosamples as a sort of collaboration between scientific communities within the same institution, country, or even overseas [12, 13]. The International Charter of Principles for Sharing Bio-specimens and Data draft the guidelines for legal and ethical principles of biosamples sharing [5, 6]. These include privacy and autonomy, freedom and openness of scientific inquiry, reciprocity, respect of intellectual contribution, and intellectual property [5]. Additionally, the actual sampling process should rely on ethical guidelines, including proper consenting of the subjects [14] and voluntary participation and withdrawal options [15], along with appropriate coding and storage of biosamples.

There is a growing concern in responsible conduct of research, in the Middle East and North Africa (MENA) region, including establishing institutional regulations, training, and schools’ curriculum development [16–18]. In Jordan, there are no guidelines that regulate the reuse, sharing, and storage of biosamples in biomedical research. In addition, the awareness and experience of biomedical researchers to share and reuse biosamples have not yet been explored in the country. Therefore, the current study had the objective to evaluate the expertise of biosamples sharing, storage, and reuse among biomedical researchers in Jordan as an example of the MENA region.

## Methodology

### Participants and study design

This study is based on a cross-sectional questionnaire. The questionnaire was approved by the Institutional Review Board (IRB) of Jordan University of Science and technology.

The study was carried out in April of 2020. Included in the study were local biomedical researchers representing most Jordanian Universities. Excluded from the study were researchers with no publication within the past three years. This study followed a convenience sampling technique where the Jordanian biomedical researchers were contacted via their official academic email addresses. The consent form was built in the questionnaire, and the subjects were not allowed to fill the self-administered questionnaire until accepting the electronic consent form. Study aims were explained to the participants, and more clarifications were provided upon request via email. Responses were recorded anonymously via online survey using Google forms.

The researcher used G*Power software version 3.1.9.7, Universitat Kiel, Germany, to calculate the sample size. A 0.05 significance level, a power of 0.90, and a medium effect size of 0.30 required the minimum number of subjects to be 204. We have distributed 496 surveys (248 of each gender). A total of 196 subjects filled the survey, representing 96.1% of the target number.

### Study instrument

The questionnaire was developed to assess the practices of biomedical researchers regarding sharing, storing, and reusing biosamples. The questionnaire was divided into two sections: demographic/general information and biosamples management practices. The available data includes gender, age, employment sector, researcher’s specialty, professional position (academic rank), number of publications, and primary funding sources. We measured different Jordanian biomedical researchers’ practices through several questions, including storing, reusing samples in future research projects, and sharing biosamples locally and outside Jordan. Furthermore, the possibility of biosamples sharing and reusing in the original consent form was also investigated. All the survey questions were presented in the Result section. The survey questions were optional, and the participants had the choice to skip any question they did not want to answer. The study questionnaire is provided as S1 File.

The study questionnaire was drafted by the research team, and was then, revised a team of experts including specialists in public health, medical laboratory sciences/biosamples handling, research ethics and data confidentiality. Questionnaire was pilot tested in 15 participants to ensure quality and comprehensibility. Additionally, subjects from the pilot sample were asked to provide comments about how they understood each survey item to ensure content clarity and comprehension. Pilot samples were omitted from the final analysis. The reliability coefficient for all items of the study was >0.65. As for validity, the study survey was face validated via review by experts in the field, including senior researchers in biology, pharmacology, public health, and research ethics.

### Statistical analysis

Data were expressed using frequency distribution for categorical variables and mean ± SD for continuous variables. Crosstabs using Pearson’s Chi square test were used to examine demographic variables according to their practices. A *P-*value < 0.05 was considered significant. Statistical analyses were conducted using SPSS version 23 (IBM Corporation, NY, USA).

## Results

The response rate was 39.5%, corresponding to 196 out of 496 distributed surveys. The Average age of respondents was 40.0 (95% C. I. 38.5–41.4) years old. Table 1 shows the frequencies of different demographic factors. Female researchers represented 42.3% (n = 83), while male researchers accounted for 57.9% (n = 113) of responses. About two-thirds of the respondents worked in public institutions and health-related disciplines such as medicine, dentistry, pharmacy, and nursing. The Academic ranks of respondents were MSc holder/ lecturer (15%), assistant professors (39%) and associate professors (26%), and professors (17.5%). More than one-third of the participants had more than 15 publications. The sources of the fund were from the academic institutions (50%), external (24.5%), and personal (self-fund: 11.5%).

**Table 1:** Demographic data of the respondent researchers (N = 196).

Variables	Categories	N (%)
**Gender**	Male	113 (57.9)
	Female	83 (42.3)
**Employment Sector**	Public	126 (64.3)
	Private	70 (35.7)
**Specialty**	Biological Sciences	69 (34.4)
	Health-Related	126 (64.6)
**Number of publications**	<15	123(62.8)
	15–30	36(18.4)
	>30	37 (18.9)
**Academic Rank**	MSc Holder/Lecturer	30 (15.0)
	Assistant Professor	78 (39.0)
	Associate Professor	52 (26.0)
	Professor	35 (17.0)
**Source of fund**	External/Industry	49 (24.5)
	Academic Institutions	101 (50.5)
	Personal	23 (11.5)

Table 2 shows practices of Jordanian researchers regarding storage, sharing, analysis, and reuse of biosamples. Concerning storage, most respondents (82.9%, n = 155) were applying coding, while 17.1% (n = 32) used the participants’ names to label the samples. About 61.7% (n = 119) reported that they have ever shared biosamples with other researchers from Jordan, whereas 47.2% (n = 92) have ever shared them with researchers from outside Jordan. Besides, about 41.3% (n = 81) reported analyzing biosamples outside of Jordan, including collaborations with researchers outside Jordan or commercial outsourcing analysis. Types of analysis were in the range of genetic and bioinformatic analysis. 55.4% (n = 108) of respondents mentioned that they had reused biosamples for future projects. Correspondingly, 63.9% (n = 124) of the researchers pointed out the possibility of future reusing the samples, sharing, and analyzing samples outside Jordan in the consent form. Essentially, 36.1% (n = 70) of researchers did not explain the possibility of future reuse of biosamples in the consent form. Among them, 15% (n = 29) reused the biosamples. Similarly, the possibility of biosamples sharing in the consent form has not been explained by 42.0% (n = 81). Among them, 17.8% (n = 34) shared the samples with other researchers.

**Table 2:** Bio-samples storage, reanalysis, and sharing among researchers in Jordan.

Variables	Categories	N (%)
**How do you store the subject’s bio-samples?**	Coded	155 (82.9)
	Using full names	32 (17.1)
**Have you ever shared the collected bio-samples with other local researchers?**	Yes	119 (61.7)
	No	74 (38.3)
**Have you ever shared the collected bio-samples with other researchers outside of Jordan?**	Yes	92 (47.2)
	No	103 (52.8)
**Have you ever sent the collected bio-samples to be analyzed outside Jordan?**	Yes	81 (41.3)
	No	115 (58.7)
**Have you ever reused stored bio-samples in future research projects?**	Yes	108 (55.4)
	No	87 (44.6)
**Do you explain the possibility of future re-use of bio-samples in the consent form?**	Yes	124 (63.9)
	No	70 (36.1)
**Do you explain the possibility of bio-samples sharing in the consent form?**	Yes	112 (58.0)
	No	81 (42.0)
**Do you explain the possibility of bio-samples analysis outside of Jordan in the consent form? **	Yes	103 (53.1)
	No	91 (46.9)

Tables 3 and 4 shows the cross-tabulation analysis of different responses of Jordanian biomedical researchers along with the relation to the various demographic factors. Storing of biosamples using coding versus the full names was significantly associated with the source of funds (P<0.01). Researchers with self-funding seem to use full names more frequently when storing biosamples than researchers with funding from external/institutional sources. Asking researchers if they have ever shared collected biosamples with other local researchers revealed significant differences with academic rank, gender, employment sector, and the number of publications (P<0.05). Additionally, sharing biosamples outside Jordan was significantly associated with gender, the number of publications, and academic rank (P<0.05). Being male, having > 30 publications, and with a high academic position (professor or associate professor) were more likely to share biosamples with local or overseas researchers. However, rank and funding are not correlated and was not worth comparing directly. Working in a private university was associated with sharing biosamples with local researchers. Concerning the analysis of samples outside of Jordan, being male and having more publications were significantly related to this practice (P<0.05). Finally, explaining the possibility of analysis of biosamples outside Jordan was associated with the number of publications and academic rank. Being a professor with more publications was more likely to explain the possibility of analyzing biosamples outside Jordan.

**Table 3:** Storage, analysis, and sharing of bio-samples among researchers in Jordan N (%), Crosstab analysis.

Questions	Sub-category	Gender			Number of Publications in International Journals				Employment Sector			Specialty		
		Male	Female	*P*-value	<15	15–30	>30	*P*-Value	Governmental	Private	*P*-value	Biology	Health Related	*P*-value
**How do you store the subject’s biosamples?**	Coded	89 (83.2)	66 (82.5)	0.903	97 (81.5)	28 (84.8)	30(85.7)	0.800	102 (84.3)	53 (80.3)	0.488	54(80.6)	101(84.2)	0.543
	Using full names	18 (16.8)	14 (17.5)		22 (18.5)	5 (15.2)	5(14.3)		19 (15.7)	13 (19.7)		13(19.4)	19(15.8)	
**Have you ever shared the collected biosamples with other local researchers?**	No	35 (31.3)	39 (48.1)	**0.017**	54 (44.6)	12 (33.3)	8 (22.2)	**0.042**	55 (44.7)	19 (27.1)	**0.016**	30(42.9)	44(35.8)	0.330
	Yes	77 (68.8)	42 (51.9)		67 (55.4)	24 (66.7)	28 (77.8)		68 (55.3)	51 (72.9)		40(57.1)	79(64.2)	
**Have you ever shared the collected biosamples with other researchers from outside Jordan?**	No	53 (46.5)	50 (61.7)	**0.036**	77 (62.6)	17 (48.6)	9 (24.3)	**0.000**	65 (52.0)	38 (54.3)	0.759	38(55.1)	65(51.6)	0.641
	Yes	61 (53.5)	31 (38.3)		46 (37.4)	18 (51.4)	28 (75.7)		60 (48.0)	32 (45.7)		31(44.9)	61(48.4)	
**Have you ever sent the collected biosamples to be analyzed outside Jordan?**	No	58 (50.9)	57 (69.5)	**0.009**	83 (67.5)	19 (52.8)	13 (35.1)	**0.002**	70 (55.6)	45 (64.3)	0.234	37(52.9)	78(61.9)	0.218
	Yes	56 (49.1)	25 (30.5)		40 (32.5)	17 (47.2)	2 4(64.9)		56 (44.4)	25(35.7)		33(47.1)	48(38.1)	
**Have you ever reused stored bio-samples in future research projects?**	No	45 (39.8)	42 (51.2)	0.114	63 (51.6)	12 (33.3)	12 (32.4)	**0.039**	53 (42.1)	34(49.3)	0.333	31(44.3)	56(44.8)	0.945
	Yes	68 (60.2)	40 (48.8)		59 (48.4)	24 (66.7)	25 (67.6)		73 (57.9)	35(50.7)		39(55.7)	69(55.2)	
**Do you explain the possibility of future re-use of bio-samples in the consent form?**	No	38 (33.9)	32 (39.0)	0.465	48 (39.7)	12 (33.3)	10 (27.0)	0.348	45 (36.3)	25(35.7)	0.936	29(41.4)	41(33.1)	0.244
	Yes	74 (66.1)	50 (61.0)		73 (60.3)	24 (66.7)	27 (73.0)		79 (63.7)	45(64.3)		41(58.6)	83(66.9)	
**Do you explain the possibility of bio-samples sharing in the consent form?**	No	47 (42.0)	34 (42.0)	0.999	56 (46.7)	13 (36.1)	12 (32.4)	0.226	53 (42.4)	28(41.2)	0.869	30(42.9)	51(41.5)	0.854
	Yes	65 (58.0)	47 (58.0)		64 (53.3)	23 (63.9)	25(67.6)		72(57.6)	40(58.8)		40(57.1)	72(58.5)	
**Do you explain the possibility of bio-samples analysis outside of Jordan in the consent form?**	No	51 (45.5)	40 (48.8)	0.655	68 (56.2)	13 (36.1)	10(27.0)	**0.003**	57(46.0)	34(48.6)	0.727	32(45.7)	59(47.6)	0.802
	Yes	61 (54.5)	42 (51.2)		53 (43.8)	23 (63.9)	27(73.0)		67(54.0)	36(51.4)		38(54.3)	65(52.4)	

**Table 4:** Storage, analysis, and sharing of bio-samples among researchers in Jordan N (%), Crosstab analysis.

Question	Sub-category	Academic Rank					Source of fund			
		MSc Holder / Lecturer	Assistant professor	Associate professor	Professor	*P*-value	External/industry	Institutional	Self	*P*-value
**How do you store subject’s biosamples?**	Coded	22 (78.6)	62 (82.7)	41 (82.0)	30(88.2)	0.781	41(89.1)	86(86.9)	12(60.0)	**0.006**
	Using full names	6 (21.4)	13 (17.3)	9 (18.0)	4(11.8)		5(10.9)	13(13.1)	8(40.0)	
**Have you ever shared the collected biosamples with other local researchers?**	No	16 (53.3)	34 (44.7)	16 (30.8)	8(22.9)	**0.030**	14(29.2)	43(42.6)	9(40.9)	0.283
	Yes	14 (46.7)	42 (55.3)	36 (69.2)	27(77.1)		34(70.8)	58(57.4)	13(59.1)	
**Have you ever shared the collected biosamples with other researchers from outside Jordan?**	No	25 (83.3)	43(55.1)	27 (52.9)	8(22.2)	**<0.0001**	20(40.8)	57(57.0)	14(60.9)	0.127
	Yes	5 (16.7)	35(44.9)	24 (47.1)	28(77.8)		29(59.2)	43(43.0)	9(39.1)	
**Have you ever sent the collected biosamples to be analyzed outside Jordan?**	No	22 (73.3)	54(69.2)	30 (57.7)	9(25.0)	**<0.0001**	25(51.0)	61(60.4)	15(65.2)	0.427
	Yes	8 (26.7)	24(30.8)	22 (42.3)	27(75.0)		24(49.0)	40(39.6)	8(34.8)	
**Have you ever reused stored bio-samples in future research projects?**	No	16 (53.3)	39(50.6)	23 (44.2)	9(25.0)	0.053	25(51.0)	41(41.0)	12(52.2)	0.400
	Yes	14 (46.7)	38(49.4)	29 (55.8)	27(75.0)		24(49.0)	59(59.0)	11(47.8)	
**Do you explain the possibility of future re-use of bio-samples in the consent form?**	No	10 (33.3)	29(38.2)	19 (36.5)	12(33.3)	0.948	18(36.7)	35(35.4)	10(43.5)	0.767
	Yes	20 (66.7)	47(61.8)	33 (63.5)	24(66.7)		31(63.3)	64(64.6)	13(56.5)	
**Do you explain the possibility of bio-samples sharing in the consent form?**	No	15 (51.7)	30(39.5)	24 (46.2)	12(33.3)	0.423	19(39.6)	44(44.0)	11(47.8)	0.786
	Yes	14 (48.3)	46(60.5)	28 (53.8)	24(66.7)		29(60.4)	56(56.0)	12(52.2)	
**Do you explain the possibility of bio-samples analysis outside of Jordan in the consent form?**	No	18 (60.0)	39(51.3)	25 (48.1)	9(25.0)	**0.022**	17(35.4)	49(49.0)	14(60.9)	0.104
	Yes	12 (40.0)	37(48.7)	27 (51.9)	27(75.0)		31(64.6)	51(51.0)	9(39.1)	

## Discussion

The current study intended to evaluate the practices of Jordanian biomedical researchers regarding sharing, storage, and reuse of biosamples. While sharing and reusing biosamples are beneficial to biomedical research [2–4]. However, it can be associated with ethical challenges such as autonomy, confidentiality, and discrimination [6, 7, 10]. Respecting participants’ autonomy can be achieved via re-consenting or the use of open consent, as in the case of biobanks [19]. Therefore, biosamples sharing and reuse should be as open as possible and restricted as necessary, according to the ultimate needs, and follow ethical regulations and norms.

Current results showed that sharing and reusing biosamples is common among Jordanian researchers. The sharing and reusing of bio-samples is becoming increasingly important to many disciplines for the integrity of science (i.e., replication) and the development of synthetic data products that allow existing data to be applied to new problems [20]. Besides, saving time and efforts of recruitments [2] and enhancing the sample size will ultimately improve the statistical power and analysis [3, 4].

One of the conducted studies in the USA showed that most patients are willing to share their data and biospecimens for research and appreciate being asked about their data and biospecimen sharing preferences [21]. A recent study from Jordan investigated the perceptions and concerns of biomedical researchers about biomedical data sharing [22]. Another study found that the public from Jordan was very positive regarding sharing their biosamples to advance biomedical research in the country [23].

However, there is a growing understanding of the risks of sharing biosamples and associated data [9]. For example, respect for a person’s autonomy and privacy can be breached by sharing and reusing biosamples [5, 6]. Disclosure of identifying biomedical data can stigmatize or discriminate individuals and populations [24]. Two studies from Nigeria [25] and South Africa [26] reported public support for the reuse of biosamples based on the condition that appropriate structures should be considered to safeguard the welfare of participants. Therefore, sharing and reuse of biosamples should be regulated to protect human research participants.

In this study, one of the findings showed that most responders agreed that storing biosamples should be coded, and a substantial fraction (17.1%) of responders preferred using the participant’s name when keeping the biosamples. In Jordan, biobanks are limited with only one biobank restricted to biospecimens from cancer patients [27]. On the other hand, regulations regarding storage of human biospecimens for research purposes are also lacking [18, 28]. Thus, biospecimens are usually stored in research laboratories that belong to a single or a team of researchers. The standard procedure of human biospecimens should include coding and appropriate labelling. The label should be able to withstand all potential transportation and storage conditions. The procedure of storing human biospecimens should also be approved by the institutional review boards. Biosample coding/encryption is highly recommended as indicated in the Human Research Protections guideline / USA [29] and International Charter of principles for sharing bio-specimens [6] as it provides some privacy protection.

In biomedical research, the subject’s name and personal information that could be used to identify research participants should not be used in the stored bio-samples as this is considered an invasion of the participants’ privacy. Besides, if used, anonymization may preclude any influence the donors have on the use of their samples. However, the complete anonymization of bio-samples should be avoided, based on the principle that this would make it impossible to add relevant data as science progresses. It precludes re-contacting donors and data subjects to communicate them as future medical discoveries may benefit them [30]. Thus, although anonymization might provide an adequate level of privacy protection, it is an imperfect solution.

The results showed that sharing biosamples with local researchers was 14% higher than sharing with researchers abroad. Additionally, there was a preference to send the collected bio-samples to be analyzed abroad than locally.

In the context of biosamples sharing across national borders with a broader research community, multiple regulations and international ethical recommendations were made to safeguard bio-samples against unintentional misuse. There are also concerns about sharing research benefits fairly and acceptably to participants, communities, and funders [31]. For example, the European Directive on Data Protection [32] mandates strict protection of personally identifiable data, including research results. However, the legal requirement to regulate biosamples sharing and analysis outside the country is lacking here in Jordan.

At this point, it is recommended to investigate the perspective of the general population in Jordan. A study from Egypt found that most participants preferred to have their samples analyzed in Arab states than other countries such as Europe and the USA [30, 31]. Factors to account for the unwillingness of those participants to share bio-samples with the western world might revolve around issues of confidentiality, commodification of the samples, cultural or religious values, and significant concerns that once bio-sample leave the country, it might be more challenging to provide oversight on the types of research performed on them [30]. Another study in Egypt showed that the participants have concerns regarding sharing their samples across borders or with pharmaceutical companies [32]. Although sending biosamples to be analyzed by international commercial laboratories might not be associated with ethical issues when confidentiality is maintained, it seems that sending biosamples to be analyzed overseas is not welcomed in the Arab culture [33, 34].

In the current study, the majority (55%) of researchers practiced reusing stored biosamples in various research projects without taking subjects’ consent for biosample reuse (36.1%). In biobanks, biosamples are used in multiple projects to implement what is currently known as “broad consent” to avoid re-consenting in every use and permit researchers for an unspecified range of future research subjects [28, 35, 36]. On the other hand, the issue of consent, re-consent, or the context in which permission is obtained seems to have no ethical concerns [37].

In Jordan, the informed consent process and the adequacy of the consent forms were addressed in previous studies [38–40]. In Saudi Arabia, informed consent was reported as a significant ethical issue in biobanking [41]. Challenges regarding informed consent were also reported in other countries from MENA, such as Sudan, Tunisia, Qatar, Lebanon, Iran, and Egypt [40, 42–45]. Thus, the quality of the research consenting process needs improvement in Jordan and MENA countries.

The findings of our study showed that sharing and reusing biosamples were associated with academic ranks, gender, employment sector, and the number of publications (P < 0.05). More specifically, the attitude towards biosamples sharing increases with higher academic ranks, where those at full professor reported a higher rate of ever sharing their research samples (77.8%). This might be expected as senior researchers have gained more experience, access to more funds, and international collaborations and networking than junior researchers (Assistant professors). Being a male and working in a private institution were more likely to share biosamples, which could be due to the lack of consent and IRB committees in private universities, in addition to the shortage of governmental funds. These are only speculations as we do not have data in the present study regarding who obtained IRB approvals and funding challenges but might be interesting for future research. Concerning the gender factor, a German study showed that females were less willing to share data than males [46], similar to that reported in Jordan, which could be due to the differences in the privacy behavior between men and women [47]. On the other hand, a study that was conducted on cancer patients, females were more willing to share their medical records data for research purposes [48]. Requirements for collaborating and sharing specimens among researchers were also found to vary according to gender [49]. Male researchers were reported to emphasize on compliance with institutional and governmental policies whereas female researchers to emphasize data sharing policies [49].

The current study choice of 15 scholarly publications as a cut-off value was based on the approximate average number of peer-reviewed articles required to fulfill the academic eligibility promotion to full professor in the Jordanian universities.

The current study highlights the need for development of proper national regulation for sharing of biosamples. This is essential to provide guidance to the researchers and to avoid research misconduct and breaching the confidentiality and privacy of research participants. The results could help in that aspect showing the gabs in the current practices of researchers in Jordan, as an example of the MENA region. However, we should note some limitations in this study—first, the relatively small sample size. Second, the low response rate (39.5%). Third, although an equal number of each gender was invited to participate in the study, more male participants (57.9%) filled the survey than female participants (42.1%). Finally, we did not collect information regarding types of biosamples. Thus, confirming our findings in a larger sample size and more female participants is strongly recommended. In addition, the inclusion of the type of biosamples in future studies can add different angles to the current study findings.

In conclusion, sharing and reusing of biosamples is common among Jordanian researchers in the biomedical field both nationally and internationally, where the majority of researchers explained the possibility of reusing and sharing biosamples in the consent form. Sharing and reusing biosamples were associated with gender, the number of publications in peer-reviewed international journals, and academic rank. The current study provides information indicating the need for new regulations, guidelines, and training regarding the management of biosamples and consent processes in research to protect research participants and maintain ethically sound research. These include the utilization of the current international ethical and governance frameworks and sample access committees.

## Supporting information

S1 Data (XLS)

S1 File (DOCX)
