# Supplementary material for: Sharing, reuse, and storage of biosamples among biomedical researchers in Jordan: Practice and concerns
Source: PLoS One. 2022 Apr 28;17(4):e0267552. doi: 10.1371/journal.pone.0267552 (PMC9049300; doi:10.1371/journal.pone.0267552)
Supplement: S1 File — (DOCX) [file pone.0267552.s002.docx]

Supplementary

Part I: Demographic and Personal Information

Gender

1. Male
2. Female

Employment Sector

1. Public
2. Private

Specialty

1. Biological Sciences
2. Health-Related

Number of Publications in International Journals

1. <15
2. 15-30
3. >30

Academic Rank

1. MSc Holder/ Lecturer
2. Assistant Professor
3. Associate Professor
4. Professor

Source of fund

1. External/Industry
2. Academic Institutions
3. Personal

Part II: Bio-Samples storage, reuse and sharing among researchers in Jordan.

How do you store the subject’s bio-samples?

1. Coded
2. Using full names

Have you ever shared the collected bio-samples with other local researchers?

1. Yes
2. No

Have you ever shared the collected bio-samples with other researchers outside of Jordan?

1. Yes
2. No

Have you ever sent the collected bio-samples to be analyzed outside Jordan?

1. Yes
2. No

Have you ever reused stored bio-samples in future research projects?

1. Yes
2. No

Do you explain the possibility of future re-use of bio-samples in the consent form?

1. Yes
2. No

Do you explain the possibility of bio-samples sharing in the consent form?

1. Yes
2. No

Do you explain the possibility of bio-samples analysis outside of Jordan in the consent form?

1. Yes
2. No
